# Blog and Podcast Watch: Pediatric Emergency Medicine

**DOI:** 10.5811/westjem.2016.6.30193

**Published:** 2016-07-21

**Authors:** Fareen Zaver, Michael Hansen, Evan Leibner, Andrew Little, Michelle Lin

**Affiliations:** * George Washington University, Department of Emergency Medicine, Washington, DC; † Christiana Care Health System, Department of Emergency Medicine, Newark, Delaware; ‡ Stony Brook University Hospital, Department of Emergency Medicine, Stony Brook, New York; § Doctors Hospital, Department of Emergency Medicine, Columbus, Ohio; ¶ University of California, San Francisco, Department of Emergency Medicine, San Francisco, California

## Abstract

**Introduction:**

By critically appraising open access, educational blogs and podcasts in emergency medicine (EM) using an objective scoring instrument, this installment of the ALiEM (Academic Life in Emergency Medicine) Blog and Podcast Watch series curated and scored relevant posts in the specific areas of pediatric EM.

**Methods:**

The Approved Instructional Resources – Professional (AIR-Pro) series is a continuously building curriculum covering a new subject area every two months. For each area, six EM chief residents identify 3–5 advanced clinical questions. Using FOAMsearch.net to search blogs and podcasts, relevant posts are scored by eight reviewers from the AIR-Pro Board, which is comprised of EM faculty and chief residents at various institutions. The scoring instrument contains five measurement outcomes based on 7-point Likert scales: recency, accuracy, educational utility, evidence based, and references. The AIR-Pro label is awarded to posts with a score of ≥26 (out of 35) points. An “Honorable Mention” label is awarded if Board members collectively felt that the posts were valuable and the scores were > 20.

**Results:**

We included a total of 41 blog posts and podcasts. Key educational pearls from the 10 high quality AIR-Pro posts and four Honorable Mentions are summarized.

**Conclusion:**

The *WestJEM* ALiEM Blog and Podcast Watch series is based on the AIR and AIR-Pro series, which attempts to identify high quality educational content on open-access blogs and podcasts. Until more objective quality indicators are developed for learners and educators, this series provides an expert-based, crowdsourced approach towards critically appraising educational social media content for EM clinicians.

## BACKGROUND

With the rapid rise in the number of social media educational content on blogs and podcasts, especially in emergency medicine (EM),^1^ there has only been preliminary progress in helping educators and learners identify quality resources.^2^–^4^ In 2008, the Accreditation Council for Graduate Medical Education endorsed a decrease in synchronous conference experiences for EM residency programs by up to 20% in exchange for asynchronous learning termed Individualized Interactive Instruction (III).^5^ Residency programs, however, were often unsure how to identify quality online resources specifically for asynchronous learning and III credit.

To address this need, the Approved Instructional Resources (AIR) Series^6^ and AIR-Pro Series were created in 2014 and 2015, respectively, by Academic Life in Emergency Medicine (ALiEM) to help EM residency programs identify quality online content specifically on social media. Using an expert-based, crowdsourced approach, these two programs identify trustworthy, high-quality, educational blog and podcast content. The intended audience for the AIR series is EM junior residents, and the AIR-Pro Series is geared toward the EM advanced practitioner. This ALiEM Blog and Podcast Watch series on *WestJEM* presents annotated summaries from the AIR and AIR-Pro Series.

This installment from the AIR-Pro Series summarizes the best scoring social media educational resources on specific topics within pediatric EM.

## METHODS

### Question Identification

The AIR-Pro series is a continuously building curriculum covering a new subject area every two months. For each area, six EM chief residents from different U.S. residency programs on the ALiEM-Pro Editorial Board identify 3–5 focused, advanced-level clinical queries within the featured subject area. The topics for this installment included the following:

1. Pediatric arrhythmias
2. Procedural sedation in pediatrics
3. The neonate in distress
4. Toddlers with a limp
5. Pediatric syncope

### Inclusion and exclusion criteria

A broad range of resources were identified using the custom EM search engine FOAMsearch.net, which extracts content from >300 blogs/podcasts relevant to EM as of February 2014.^7^ We included blog posts and podcasts written in English and identified by key search terms for our scoring by our expert panel. We excluded journal articles from the list.

### Scoring

Extracted posts were scored by eight reviewers from the AIR-Pro Editorial Board, which is comprised of EM core faculty and chief residents from various U.S. institutions. The eight reviewers included five chief residents from the AIR-Pro Editorial Board as well as three EM faculty educators, which included one board-certified pediatric EM educator and one pediatric EM fellow.

The scoring instrument contains five measurement outcomes using 7-point Likert scales: recency, accuracy, educational utility, evidence based, and references (Table). The scores for each post were evenly averaged over the eight reviewers. There were minimal disagreements regarding the scores, and the total for the five measured outcomes were tallied together to determine a final score out of 35 total points. We have sought to optimize content and response process validity of the scoring tool and assessment quizzes by expert development, review, and pilot testing. Although the inter-rater reliability of the scoring tool has not been performed, the tool was constructed to be simple and unambiguous similar to that of the AIR series this was modeled after.^6^

### Data Analysis

An AIR-Pro endorsement is given to posts with a score of ≥26 (out of 35) points. Depending on the redundancy of the highest scoring posts, the best of these are then selected to address each preselected topic. An “Honorable Mention” label is also given to posts specifically felt to be worthwhile, accurate, unbiased, and educationally valuable for advanced clinicians by consensus of the AIR-Pro Board members. These posts must have scored ≥20 (out of 35) points.

## RESULTS

We initially included a total of 41 blog posts and podcasts. Key educational pearls from the 10 high quality AIR-Pro posts and four Honorable Mentions are summarized.

### AIR-Pro Content

1. Greene A, Patel S. ECGs: Long QT and Brugada. PEM Academy. (Sept 18, 2014) http://www.pemacademy.com/ecgs-long-qt-and-brugada/

This 10-minute video presentation by Gopwani, Patel, Greene, and Chapman from the Pediatric EM Academy blog covers the pathologies related to Long QT Syndrome (exercise, stress, electrolyte abnormalities, etc) and Brugada Syndrome. Case-based content frames the discussion regarding the various pathologies, emergency department (ED) management, and patient disposition.

Long QT Syndrome take-home points: Pediatric patients with a QTc >470 msec should be seen emergently by a cardiologist in the ED. Long QT syndrome is typically either caused by a congenital abnormality or it is acquired (e.g. electrolyte and substance induced). Consider long QT syndrome as an etiology when exertion or emotional stress precedes syncope or seizures.

Brugada Syndrome take-home points: The electrocardiogram (ECG) in Brugada Type 1 appears as a “pseudo” right bundle branch block, coupled with ST segment elevation in V1, V2, and V3. The ECG in Brugada Types 2 and 3 has a saddleback-shaped pattern with a positive or biphasic T wave seen in V1, V2, and V3. The difference between Brugada type 2 and 3 is the amount of ST elevation with Type 2 having ≥1 mm of elevation and Type 3 having <1 mm of elevation.

2. Fox S. Brugada in Children. *Pediatric EM Morsels*. (Sept 11, 2015) http://pedemmorsels.com/brugada-in-children/

This blog discusses the diagnosis and management of Brugada Syndrome. It focuses on the special considerations of Brugada Syndrome in the pediatric population.

Take-home points: Febrile illnesses can trigger arrhythmias in pediatric patients with underlying Brugada Syndrome. Children with Brugada Syndrome are more likely to present with complications of monomorphic ventricular tachycardia in comparison to older patients who present with ventricular fibrillation. Although the best available treatment option is an automatic implantable cardioverter-defibrillator (AICD), the decision to place one requires a careful risk-benefit discussion because there is a higher rate of AICD complications in children.

3. Forbes E. Syncope Sunday 4: Syncope ECGs. Don’t Forget the Bubbles. (March 15, 2015) http://dontforgetthebubbles.com/syncope-ecgs/

This post discusses the cardiac causes of syncope in pediatric patients including hypertrophic obstructive cardiomyopathy (HOCM), arrhythmogenic right ventricular dysplasia (ARVD), Wolff-Parkinson-White syndrome, Brugada syndrome, long QTc, and catecholaminergic polymorphic ventricular tachycardia (CPTV). A classic ECG is provided for each syndrome, except CPTV.

Take-home points: The clinician must maintain a high index of suspicion for subtle ECG findings in the setting of syncope in children. Because HOCM is the leading cause of sudden death in young athletes, look for high left ventricular voltage, Q waves in the inferior-lateral leads, and left atrial enlargement in the setting of a concerning story. ARVD is the second most common cause of sudden death in young athletes. Look for the epsilon waves, T wave inversions in V1–V3, and prolonged S-wave upstroke.

4. Tat S. ECGs: Heart Block and Sick Sinus. PEM Academy. (July 17, 2014) http://www.pemacademy.com/ecgs-heart-block-and-sick-sinus/

This is a 12-minute video presentation covering heart block and sick sinus syndrome created by Gopwani, Patel, Greene, and Chapman. Heart blocks and sick sinus conditions are discussed through real-life cases discussing possible patient presentations, acute management, and appropriate disposition for these patients.

Take-home points: Reading the ECG using the same methodical approach every time will aid in identifying subtle atrioventricular (AV) blocks. Second-degree AV blocks typically happen in a set pattern such as 4:3 and 3:2. All patients with a third-degree AV block require an immediate cardiology consultation. In contrast, only symptomatic patients with a second-degree AV block require cardiology consultation in the ED.

5. Fox S. Ketamine for Analgesia. *Pediatric EM Morsels*. (April 11, 2014) http://pedemmorsels.com/ketamine-analgesia/

This blog post highlights current practices using ketamine as a primary pain medication in the pediatric population. Discussion points include indications, dosages, and barriers to use.

Take-home points: Ketamine should be considered as a core medication in basic healthcare systems. Ketamine is safe and effective, has a rapid onset, low risk for respiratory depression, and requires little monitoring when administered at analgesic doses (0.1–0.3 mg/kg IV or 0.5–1 mg/kg IM).

6. Sobo B. Ketamine. *PEM Currents*. (July 24, 2013) http://www.pemcincinnati.com/podcasts/?p=60

This 8-minute podcast covers the basics of ketamine use in the pediatric population. It focuses on the benefits of ketamine including rapid onset, low side-effect profile, and ease of administration. Discussion points also include parental education regarding tachycardia and hypertension, as well as factors that increase a child’s risk of adverse reactions.

Take-home points: Ketamine provides both amnestic and analgesic properties safely and efficiently. Younger patients metabolize ketamine at a faster rate and so may require more frequent re-dosing. Skillfully educating families of the possible effects prior to its use will decrease parental anxiety.

7. Radwine Z. The Sick Neonate. *EmDocs*. (Aug 12, 2014) http://www.emdocs.net/sick-neonate/

This blog post reviews the initial approach to the evaluation, management, and resuscitation of the sick neonate. The post covers the topics of sepsis, congenital heart diseases, and metabolic disturbances. It includes a stepwise approach to the history and physical exam, as well as a mnemonic to help remember the differential diagnosis for a ill-appearing neonate.

Take-home points: The differential diagnosis for the ill-appearing neonate can be remembered using the mnemonic THE MISFITS and NEO SECRETS. Neonates in extremis without a fever have a congenital heart disease until proven otherwise. A pink baby in shock needs prostaglandin (PGE1) and milrinone. In contrast, a cyanotic baby in shock needs epinephrine or norepinephrine.

8. Partyka C. A Knackered Neonate… *The Blunt Dissection* (July 20, 2013) http://thebluntdissection.org/2013/07/a-knackered-neonate/

This blog post covers an initial approach to the evaluation and management of an ill-appearing neonate. The case unfolds as you read with scaffolded questions for you to consider before revealing the answers. In this case, a hypoxic newborn presents with worsening respiratory distress despite being on nasal CPAP. The differential diagnosis is discussed, as well as neonatal intubation and troubleshooting the ventilator.

Take-home points: Neonates will become hypothermia very quickly, especially during the prolonged exposure times of endotracheal intubation. Ensure adequate external heating during the resuscitation. Troubleshooting the ventilator can be remembered using the DOPE mnemonic, which stands for Displacement of tubing, Obstruction, Pneumothorax, and Equipment failure.

9. Orman R. Pediatric Limp - Podcast: 28:22. *ER CAST*. (Feb 2, 2010) http://blog.ercast.org/pediatric-limp/

This 28-minute podcast discusses the workup for a child with a limp as well as a decision rule for distinguishing toxic synovitis from a septic arthritis of the hip. A pediatric orthopedist Dr. Adam Barmada is curbsided for a consultation to give additional pearls for dealing with a limping child.

Take-home points: Examine the entire leg of the limping child, especially a joint above and below the subjectively painful area because, for instance, knee pain may be from hip pathology (and vice versa). The Kocher Criteria for risk stratifying septic hip arthritis includes non-weight bearing status, temperature >38.5°C (101.3°F), erythrocyte sedimentation rate> 40 mm/hr, and serum white blood cell count >12 cells/mm^3^. The risk for septic arthritis is 0.2% (0 risk factors) and 97% (all four risks factors).

10. Fox S. Toddler’s Fracture. *Pediatric EM Morsels*. (Feb 1, 2013) http://pedemmorsels.com/toddlers-fracture

This blog post covers an initial approach to the evaluation and management of a toddler’s fracture. It also reviews management strategies for challenging cases, such as children with a normal workup but continue to have symptoms.

Take-home points: Toddler’s fractures are non-displaced spiral or oblique fractures of the tibia and may not be visible on initial radiographs in 13–43% of cases. Other areas that may also be initially normal on radiographs include the base of first metatarsal bone, cuboid, and calcaneus and so should be carefully palpated in toddlers with undifferentiated leg pain. A limping pediatric patient is more likely to have a fracture than a ligamentous strain even with a negative radiograph.

### Honorable Mention Content

1. Orman R. The Toxic Neonate. *ER CAST*. (May 26, 2010) http://blog.ercast.org/the-toxic-neonate/

This 37-minute podcast features an interview with Dr. Tim Horeczko, who was then a pediatric EM fellow. It was chosen as an honorable mention as it gives listeners useful information on the toxic neonate as well as a clear set of clinical priorities to work with for medical decision-making. It was determined to be a must-know resource for evaluating a sick neonate. Topics covered included the pediatric assessment triangle, hypoglycemia, intravenous fluid management, heart disease, and inborn errors of metabolism.

Take-home points: With a sick neonate, remember the pediatric assessment triangle (appearance, work of breathing, circulation) and to continue to reassess after interventions. Always check blood sugar and replete appropriately based on the different age groups: 10% dextrose 5–10 cc/kg (0–1 year old), 25% dextrose 2–4 cc/kg (1–8 years old), and 50% dextrose 1–2 cc/kg (>8 years old). In a “crashing infant” in its first month of life, if your interventions are not working, consider PGE, and if administering PGE, they will likely require intubation due to the risk for apnea.

2. Helman A. Episode 35: Pediatric Orthopedics Pearls and Pitfalls. *Emergency Medicine Cases*. (August 8, 2013) http://emergencymedicinecases.com/episdode-35-pediatric-orthopedics/

This 140-minute podcast was chosen as an honorable mention due to the careful explanation of the injuries they discussed. While a lengthy podcast, it discusses a broad spectrum of pediatric orthopedic conditions. Dr. Sanjay Mehta and Dr. Jonathan Pirie, both Canadian pediatric EM physicians, discuss septic arthritis, toddler’s fracture, tillaux fracture, supracondylar fractures, anterior cruciate ligament tears, non-accidental trauma, pediatric orthopedic pain management, and more.

Take-home points: Remember that in children their ligaments are stronger than their bones, making fractures more likely than sprains. To determine the difference between septic arthritis and transient synovitis, the Kocher criteria (inability to weight bear, ESR >40 mm/hr, fever, serum white cell count >12 cells/mm^3^), C-reactive protein, or ultrasound can be used. Ultimately one must use clinical judgment to differentiate the two conditions, because no one measure is perfect. Remember the CRITOE mnemonic (Capitellum, Radial head, Internal (medial) epicondyle, Trochlea, Olecranon, External (lateral) epicondyle) to avoid mistaking an ossification center for an avulsion fracture or vice versa. Comparison of the asymptomatic side is also useful to determine if there is an abnormality.

3. Radwine Z. Pediatric Syncope. *EmDocs*. (July 7, 2014) http://www.emdocs.net/pediatric-syncope/

This blog post was chosen as an honorable mention because of the practical and well-outlined list of pearls on pediatric syncope. The post discusses red flag considerations, cardiovascular causes, and concerning ECG findings.

Take-home points: Remember the 6 P’s when obtaining you syncope history in children: prodrome activities and prodrome symptoms, predisposing and precipitating factors, passerby/witness, and post-ictal phase. Cautionary red flag signs and symptoms include a history of congenital heart disease, no prodromal symptoms, syncope while supine or during exertion, family history of sudden cardiac death, a pathologic heart murmur, and chest pain.

4. Burns E, Nickson C, Mattu A. Hypertrophic Cardiomyopathy. *Life in the Fastlane*. (July 30, 2012) http://lifeinthefastlane.com/ecg-library/hcm/

This blog post was chosen as an honorable mention because of its in-depth review of hypertrophic cardiomyopathy (HOCM). It reviews the background, pathophysiology, and various ECG abnormalities seen with HCOM. Additionally, it includes a link to a 17-minute video presentation by Dr. Amal Mattu on the early recognition and treatments of HOCM as well as multiple brief videos from Dr. Sanjay Sharma discussing classic and subtle ECG findings in HOCM.

Take-home points: The chief abnormality associated with HOCM is left ventricular hypertrophy (LVH), occurring in the absence of any inciting stimulus such as hypertension or aortic stenosis.

Additional ECG findings are deep, narrow (dagger-like) q waves in both lateral (V5–6, I, aVL) and inferior (II, II, aVF) leads. These q waves are <40 msec (compared to those related to a myocardial infarction which are typically >40 msec). HOCM can present with arrhythmias including Wolff-Parkinson-White, atrial fibrillation, supraventricular tachycardia, premature atrial contractions, premature ventricular contractions, and ventricular tachycardia.

## CONCLUSION

Until more objective quality indicators are developed, the *WestJEM* ALiEM Blog and Podcast Watch series serves to identify educational quality blogs and podcasts for EM clinicians through an expert panel using an objective scoring instrument. These social media resources are currently curated in the ALiEM AIR and AIR-Pro Series, originally created to address EM residency needs. These resources are herein shared and summarized to help EM clinicians filter the multitude of blog posts and podcasts that are constantly being published at a rapid rate. Limitations include the fact that the search engine FOAMSearch.net may have omitted valuable websites, and the scoring instrument has not been validated. While these lists are by no means a comprehensive analysis of the entire web for these topics, this series provides a crowdsourced, expert-panel approach towards identifying some high quality, educational social media content for the EM clinician.

## Figures and Tables

**Table:** Approved Instructional Resources – Professional (AIR-Pro) series scoring instrument for blog and podcast content in pediatric emergency medicine (maximum score = 35 points).

Tier 1: Recency	Score	Tier 2: Content accuracy	Score	Tier 3: Educational Utility	Score	Tier 4: Evidence Based Medicine	Score	Tier 5: Referenced	Score
When was the blog post or podcast published?		Do you have any concerns about the accuracy of the data presented or conclusions of this article?		Are there useful educational pearls in this article for senior residents?		Does this article reflect evidence based medicine (EBM) and thus lack bias?		Are the authors and literature clearly cited?	
≥6 years ago or unknown	1	Yes, many concerns from many inaccuracies	1	Low value: No valuable pearls	1	Not EBM based, only expert opinion	1	No	1
5–6 years ago	2		2		2		2		2
4–5 years ago	3	Yes, a major concern about few inaccuracies	3	Yes, but there are only a few (1–2) valuable or multiple (>=3) less-valuable educational pearls	3	Minimally EBM based	3		3
3–4 years ago	4		4		4		4	Yes, authors and general references are listed (but no in-line references)	4
2–3 years ago	5	Minimal concerns over minor inaccuracies	5	Yes, there are several (>=3) valuable educational pearls, or a few (1–2) KEY educational pearls that every resident should know before graduating	5	Mostly EBM based	5		5
1–2 years ago	6		6		6		6		6
<1 year ago	7	No concerns over inaccuracies	7	Yes, there are multiple KEY educational pearls that residents should know before graduating	7	Yes exclusively EBM based (unbiased)	7	Yes, authors and in-line references are provided	7
